# In *Saccharomyces cerevisiae*, the molecular chaperone proteins Ssb1 and Ssb2 upregulate ABC transporter genes, and their upregulation may play a role in the release of quorum-sensing molecules that induce cell growth arrest during the diauxic shift

**DOI:** 10.3934/microbiol.2025031

**Published:** 2025-09-02

**Authors:** Yoichi Yamada, Mahiro Ota, Atsuki Shiroma, Takaki Matsuzawa

**Affiliations:** 1 Faculty of Biological Science and Technology, Institute of Science and Engineering, Kanazawa University, Kanazawa 920-1192, Japan; 2 Division of Biological Science and Technology, Kanazawa University, Kanazawa 920-1192, Japan

**Keywords:** *Saccharomyces cerevisiae*, *SSB1*, molecular chaperone, *PDR5*, ABC transporter, diauxic shift, quorum-sensing

## Abstract

In *Saccharomyces cerevisiae*, the molecular chaperone proteins Ssb1 and Ssb2 (Ssb1/2) and the cochaperone ribosome-associated complex (Zuo1 and Ssz1) localize around the ribosome tunnel exit, assisting in the maturation of nascent polypeptides. Exogenous expression of the Zuo1 C-terminus or the Ssz1 N-terminus—but not Ssb1/2—independently activates the transcription factor Pdr1 (but not Pdr3), enhances the transcription of the ATP-binding cassette (ABC) transporter genes *PDR5*, *SNQ2*, and *YOR1*, and increases pleiotropic drug resistance. Furthermore, upregulation of ABC transporter genes by *ZUO1* and *SSZ1* leads to the release of quorum-sensing molecules, which cause cell growth arrest during diauxic shifts. In this study, we examined whether *SSB1/2* are required for the expression of ABC transporter genes and the release of quorum-sensing molecules that lead to cell growth arrest during diauxic shifts. Our results show that Ssb1/2 increased the mRNA levels of *PDR5*, *SNQ2*, and *YOR1* during the late logarithmic growth phase and increased resistance to cycloheximide and fluconazole, possibly via the same pathway as Zuo1 or Ssz1. Furthermore, Ssb1/2 induced *PDR5* expression and resistance to cycloheximide and fluconazole, possibly via the same pathway as Pdr3 (but not Pdr1). In addition, it was suggested that Ssb1/2 are involved in the release of quorum-sensing molecules into the culture medium, which could signal cell growth arrest during diauxic shifts. This work provides useful knowledge regarding genetic interactions between the ribosome-associated molecular chaperone and cell growth arrest during diauxic shifts.

## 1. Introduction

In the presence of glucose, *Saccharomyces cerevisiae* grows by ethanol fermentation using glucose as a carbon source. When glucose is depleted in media, yeast cells switch from glucose fermentation to respiration by utilizing accumulated ethanol as a carbon source, which decreases translation and growth rates [1]–[3]. This conversion, known as the diauxic shift, is accompanied by major transcriptional changes in genes [1],[3].

The molecular chaperone proteins Ssb1 and Ssb2 (Ssb1/2) and the Ssb1/2 cochaperone ribosome-associated complex (RAC) localize around the ribosome tunnel exit and facilitate the de novo cotranslational folding of nascent polypeptides as they emerge from the exit [4]. Ssb1/2 differ by only four amino acids; therefore, their function is redundant [5]. Ssb1/2 are canonical Hsp70 proteins composed of an N-terminal nucleotide binding domain (NBD), a linker domain, a C-terminal substrate binding domain β (SBDβ), and a substrate binding domain α (SBDα or lid domain) [6]. Ssb1/2 bind to 60S ribosomal large subunit proteins via the C-terminus of SBDα and interact with rRNA via SBDα [6]. Double deletion of *SSB1/2* causes slow growth, cold sensitivity, hypersensitivity to the drugs hygromycin B and paromomycin, defects in glucose repression, and defects in ribosome biogenesis [7]–[10].

The RAC consists of the eukaryote-specific J-protein Zuo1 and the atypical Hsp70 homologue Ssz1 [7],[10]. Zuo1 binds to the ribosome at the tunnel exit, while Ssz1 binds to ribosomes via Zuo1 [7],[10]. RAC interacts transiently with the N-terminal NBD of Ssb1/2, stimulating ATP hydrolysis by Ssb1/2 and stabilizing the binding of the C-terminal substrate-binding domain to unfolded polypeptide substrates [11]. Zuo1 consists of an N-terminal domain (ND), which is required for heterodimerization with Ssz1; a J-domain, which stimulates ATP hydrolysis by Ssb1/2; a zuotin homology domain (ZHD), which is necessary for binding to the 60S ribosomal large subunit and the 25S rRNA; a highly charged middle domain; and a C-terminal 4-helix bundle domain, which binds to the 40S ribosomal small subunit [12]. Ssz1 contains an NBD that binds to ATP but lacks ATPase activity, and an SBDβ, which is necessary for binding to the ND of Zuo1 but is not required for normal growth [12]. Moreover, Ssz1 lacks the C-terminal SBDα lid domain. In addition, although the J-protein transiently interacts with the Hsp70 chaperone, Zuo1 and Ssz1 form an unusually stable heterodimer [13]. Therefore, Ssz1 does not appear to function as a canonical Hsp70 chaperone but instead acts as a cofactor that supports Zuo1 in regulating ATP hydrolysis by Ssb1/2 [14]. However, Zhang et al. reported that Zuo1 and Ssz1 contact nascent polypeptides, facilitating their transfer from Ssz1 to Ssb1/2 [12]. Deletion of *ZUO1* or *SSZ1* results in phenotypes similar to those resulting from deletion of *SSB1/2*, such as slow growth, cold sensitivity, high salt sensitivity, and aminoglycoside sensitivity [15],[16].

The multidrug resistance phenotype of *S. cerevisiae* is referred to as pleiotropic drug resistance (PDR). Interestingly, exogenous expression of either the Zuo1 C-terminus or the Ssz1 N-terminus—but not Ssb1/2—independently activates the Pdr1 transcription factor and enhances the transcription of the major drug efflux pump gene *PDR5* [17]–[20]. Moreover, deletion of *ZUO1* and *SSZ1* does not trigger the secretion of quorum-sensing molecules involved in growth arrest during diauxic shifts [19]. Similarly, deletion of the major drug efflux pump genes *PDR5* and *SNQ2* also fails to induce the release of quorum-sensing molecules [19]. In *S. cerevisiae*, Pdr1 and/or Pdr3 are responsible for the transcription of the drug efflux ABC transporter genes *SNQ2*, *YOR1*, *PDR5*, *PDR10*, and *PDR15* [21]–[23]. Pdr1 and/or Pdr3 bind to DNA consensus motifs, referred to as pleiotropic drug response elements (PDREs). PDREs are present in the *SNQ2*, *YOR1*, *PDR5*, *PDR10*, and *PDR15* promoter regions [21]–[23]. *PDR1* and *PDR3* display some functional redundancy, as the simultaneous deletion of both genes is necessary to cause a significant reduction in the basal expression of *PDR5* and PDR [24],[25]. Gain-of-function mutations such as *pdr1-3* and *pdr3-7* in *PDR1* and *PDR3* increase the expression of *PDR3*, *SNQ2*, *YOR1*, *PDR5*, *PDR10*, and *PDR15* to activate PDR [26]–[28].

Here, we show that Ssb1/2 upregulate the basal expression of ABC transporter genes in the late logarithmic growth phase, possibly via the same pathway as *ZUO1*, *SSZ1*, or *PDR3*. Furthermore, Ssb1/2 may contribute to the release of quorum-sensing molecules into the culture medium, which causes cell growth arrest during diauxic shifts.

## 2. Materials and methods

### 2.1. Yeast strains, plasmids, and media

All yeast strains used in this study were isogenic with FY1679-28C (MATa, ura3-52, leu2-D1, trp1-D63, his3-D200, GAL2+) [29]–[31]. To generate gene deletions, the open reading frames of *SSB1*, *SSB2*, *PDR1*, *PDR3*, *ZUO1*, or *SSZ1* were replaced with gene marker cassettes (*bleMX6*, *KanMX*, *URA3*, or *TRP1*) by a one-step gene disruption technique [29]–[32]. The yeast strains used in this work are listed in Table 1.

The centromeric plasmid pRS313 (cen, *HIS3*) was purchased from the National Bio-Resource Project, Japan. *SSB1* was amplified from genomic DNA using PrimeSTAR GXL polymerase (TaKaRa) and the following primers homologous to positions -500 and +2342: *SSB1* forward, 5′- GGCGTCGACCAGAGGAGTACACACGGGACTTGAT-3′ and *SSB1* reverse, 5′- CCGGGATCCAAAATTGGGCATTACGCCCGAAGGT-3′. The PCR product and the pRS313 plasmid were digested with *BamH*I and *Sal*I restriction enzymes. The digested PCR product was cloned and inserted into the linearized pRS313 plasmid using the DNA Ligation Kit Mighty Mix (TaKaRa) [32]. The resulting plasmid was labeled pRS313–*SSB1*.

Yeast cells were cultured in yeast peptone dextrose (YPD) media (2% glucose, 1% yeast extract, 2% bactopeptone) or synthetic complete (SC) media (0.67% yeast nitrogen base with ammonium sulfate without amino acids, 2% glucose, supplemented with all amino acids) at 30 °C.

**Table 1.** Yeast strains used.

Yeast strain	Genotype	Source or reference
FY1679-28C	*MATa ura3-52 leu2-Δ1 trp1-Δ63 his3-Δ200 GAL2+*	Yamada 2021
*ssb1Δ*	*MATa ssb1Δ*::*bleMX6 ura3-52 leu2-Δ1 trp1-Δ63 his3-Δ200 GAL2+*	Yamada et al. 2023
*ssb1Δssb2Δ*	*MATa ssb1Δ*::*kanMX ssb2Δ*::*bleMX6 ura3-52 leu2-Δ1 trp1-Δ63 his3-Δ200 GAL2+*	Yamada et al. 2023
*zuo1Δ*	*MATa zuo1Δ*::*bleMX6 ura3-52 leu2-Δ1 trp1-Δ63 his3-Δ200 GAL2+*	This study
*ssz1Δ*	*MATa ssz1Δ*::*bleMX6 ura3-52 leu2-Δ1 trp1-Δ63 his3-Δ200 GAL2+*	This study
*pdr1Δ*	*MATa pdr1Δ*::*bleMX6 ura3-52 leu2-Δ1 trp1-Δ63 his3-Δ200 GAL2+*	Onda et al. 2004
*pdr3Δ*	*MATa pdr3Δ*::*bleMX6 ura3-52 leu2-Δ1 trp1-Δ63 his3-Δ200 GAL2+*	Yamada 2021
*ssb1Δssb2Δzuo1Δ*	*MATa ssb1Δ*::*kanMX ssb2Δ*::*bleMX6 zuo1Δ*::*ura3 ura3-52 leu2-Δ1 trp1-Δ63 his3-Δ200 GAL2+*	This study
*ssb1Δssb2Δssz1Δ*	*MATa ssb1Δ*::*kanMX ssb2Δ*::*bleMX6 ssz1Δ*::*trp1 ura3-52 leu2-Δ1 trp1-Δ63 his3-Δ200 GAL2+*	This study
*ssb1Δssb2Δpdr1Δ*	*MATa ssb1Δ*::*kanMX ssb2Δ*::*bleMX6 pdr1Δ*::*ura3 ura3-52 leu2-Δ1 trp1-Δ63 his3-Δ200 GAL2+*	This study
*ssb1Δssb2Δpdr3Δ*	*MATa ssb1Δ*::*kanMX ssb2Δ*::*bleMX6 pdr3Δ*::*trp1 ura3-52 leu2-Δ1 trp1-Δ63 his3-Δ200 GAL2+*	This study

### 2.2. Spot dilution assay

To estimate the drug resistance of each strain to fluconazole or cycloheximide, a spot dilution assay was performed in triplicate, comprising two biological replicates and one technical replicate [29]–[31]. The cells from the wild-type and mutant strains were grown to the logarithmic growth phase (at an OD_600_ of 0.6–0.9) at 30 °C in YPD media. Logarithmic growth phase cultures containing the same number of cells were serially diluted 10-fold, and 3 µL aliquots of each dilution were spotted on YPD plates with and without fluconazole (Nacalai Tesque) or cycloheximide (Wako) at the indicated concentrations. The plates were then incubated at 30 °C for 7 days.

### 2.3. RNA extraction from yeast cells

Two independently derived isolates of each strain were cultured overnight in YPD medium. The overnight cultures were diluted to an OD_600_ of 0.1 and allowed to grow further at 30 °C with aeration until reaching an OD_600_ of 0.6–1.9. After recovery, the cell cultures were pelleted and washed. The cell pellets were frozen at -80 °C and used for RNA extraction [29]–[32]. Total RNA was isolated from the yeast cells using the NucleoSpin RNA kit (TaKaRa) according to the manufacturer's protocol.

### 2.4. Real-time RT-PCR

FastGene Scriptase II cDNA 5x ReadyMix (NIPPON Genetics) was used to synthesize cDNA from individual total RNA samples in duplicate. SYBR Green real-time RT-PCR for cDNA was performed using TB Green Premix Ex Taq II (TaKaRa) in a Step One Real-time PCR system (Applied Biosystems) [33]. To generate the standard curve for each primer pair, tenfold serial dilutions of cDNA from the wild-type strain were prepared. The primers used for real-time RT-PCR are listed in the Supplemental Appendix. A non-reverse-transcriptase control was used as the negative control. Endogenous *ACT1* mRNA levels were used for normalization. The normalized mRNA levels are shown relative to those in the wild-type strain.

### 2.5. Growth curves

Saturated overnight cultures of each yeast strain were diluted to an OD_600_ of 0.2 in 5 mL of SC media. The cell cultures were grown at 30 °C with shaking, and aliquots were removed periodically to measure the turbidity of the cells at 600 nm. The experiment was performed using two biological replicates and two technical replicates; data from two biological replicates are presented in Figure 5.

### 2.6. Statistical analysis

An unpaired Welch's t-test was used for statistical analysis, as shown in Figures 2 and 3. The results were considered statistically significant when p < 0.05 and p < 0.01. One-way ANOVA was used for statistical analysis, as shown in Figure 3. The results were considered statistically significant when p < 0.01.

## 3. Results

### 3.1. Ssb1/2 contribute to cycloheximide and fluconazole resistance in S. cerevisiae

**Figure 1.**
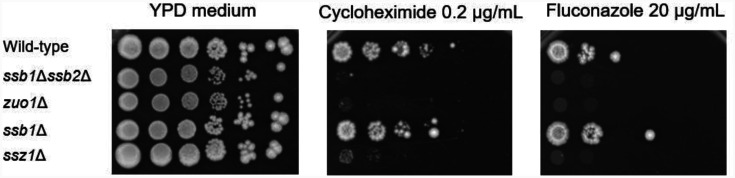
*SSB1/2* are responsible for cycloheximide and fluconazole resistance in *S. cerevisiae*. The resistance of the wild-type strain and its derivative mutant strains, *ssb1*∆*ssb2*∆, *zuo1*∆, *ssb1*∆, and *ssz1*∆, to fluconazole or cycloheximide was determined via a spot dilution assay.

Eisenman et al. reported that the overexpression of Ssb1/2 from a multicopy plasmid does not increase resistance to cycloheximide and that Zuo1 and Ssz1 can induce PDR in the absence of *SSB1/2* when they are free of ribosomes [18]. Therefore, we investigated whether the deletion of *SSB1/2* leads to increased sensitivity to cycloheximide or fluconazole. A spot dilution assay was performed to assess the resistance or susceptibility of the wild-type, *ssb1*∆*ssb2*∆, *zuo1*∆, *ssb1*∆, and *ssz1*∆ strains to cycloheximide or fluconazole. Compared with the wild-type and *ssb1*∆ strains, the *ssb1*∆*ssb2*∆, *zuo1*∆, and *ssz1*∆ strains were more susceptible to cycloheximide and fluconazole (Figure 1). These results suggest that *SSB1/2* are responsible for resistance to cycloheximide and fluconazole.

### 3.2. SSB1/2 are necessary for the upregulation of PDR5, SNQ2, and YOR1 mRNA levels in the late logarithmic growth phase

**Figure 2.**
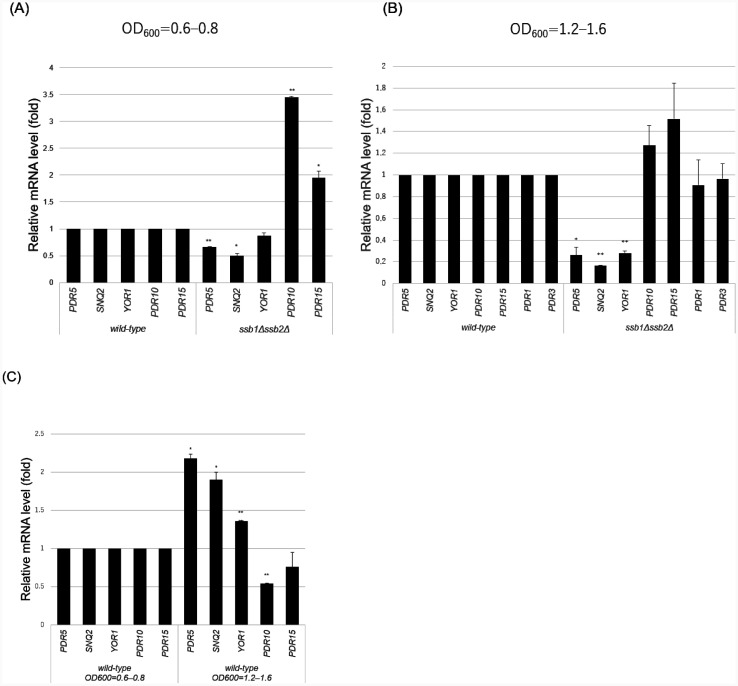
mRNA levels of ABC transporters and their regulators in the wild-type and *ssb1*∆*ssb2*∆ strains during the early and late logarithmic growth phases. (A) The relative mRNA levels of *PDR5, SNQ2, YOR1, PDR10*, and *PDR15* were determined in the wild-type and *ssb1*∆*ssb2*∆ strains at an OD_600_ of 0.6–0.8 via real-time RT-PCR. (B) Relative mRNA levels of *PDR5, SNQ2, YOR1, PDR10*, *PDR15, PDR1*, and *PDR3* were determined in the wild-type and *ssb1*∆*ssb2*∆ strains at an OD_600_ of 1.2–1.6 via real-time RT-PCR. (C) Relative mRNA levels of *PDR5, SNQ2, YOR1, PDR10*, and *PDR15* were determined in the wild-type at OD_600_ values of 0.6–0.8 and 1.2–1.6 via real-time RT-PCR. One asterisk (*) or two asterisks (**) indicate p values less than 0.05 or 0.01, respectively.

Since *SSB1/2* are responsible for cycloheximide and fluconazole resistance, we investigated whether Ssb1/2 are required for basal expression of drug efflux ABC transporter genes. Thus, we investigated the steady-state mRNA levels of the major ABC transporter genes *PDR5*, *SNQ2*, *YOR1*, *PDR10*, and *PDR15*, and their regulatory transcription factor genes *PDR1* and *PDR3*, in the wild-type and *ssb1*∆*ssb2*∆ strains via real-time RT-PCR. Although the mRNA levels of *PDR5* and *SNQ2* (but not those of *YOR1*, *PDR10*, and *PDR15*) were significantly lower in the *ssb1*∆*ssb2*∆ strain than in the wild-type strain during the early logarithmic growth phase (OD_600_ = 0.6–0.8) (p < 0.05), the differences were small (Figure 2A). During the late logarithmic growth phase (OD_600_ = 1.2–1.6), the mRNA levels of *PDR5, SNQ2*, and *YOR1* (but not those of *PDR10*, *PDR15*, *PDR1*, and *PDR3*) were significantly lower in the *ssb1*∆*ssb2*∆ strain than in the wild-type strain (Figure 2B). Furthermore, the reduction in *PDR5, SNQ2*, and *YOR1* mRNA levels was greater during the late logarithmic growth phase than during the early logarithmic growth phase. In addition, the mRNA levels of *PDR5, SNQ2*, and *YOR1* in the wild-type strain were higher during the late logarithmic growth phase than during the early logarithmic growth phase (Figure 2C).

Since cycloheximide and fluconazole are substrates of Pdr5 (but not of Snq2 or Yor1) [34],[35], the significant reduction in *PDR5* mRNA levels in the *ssb1*∆*ssb2*∆ strain can explain the sensitivity of the *ssb1*∆*ssb2*∆ mutant strain to cycloheximide and fluconazole in spot dilution assays (Figures 1 and 2). These results suggest that *SNQ2*, *YOR1*, and *PDR5* are upregulated by Ssb1/2 during the late logarithmic growth phase.

### 3.3. Ssb1/2 upregulate the mRNA levels of ABC transporters possibly via the same pathway as *Zuo1*, *Ssz1*, or *Pdr3*

**Figure 3.**
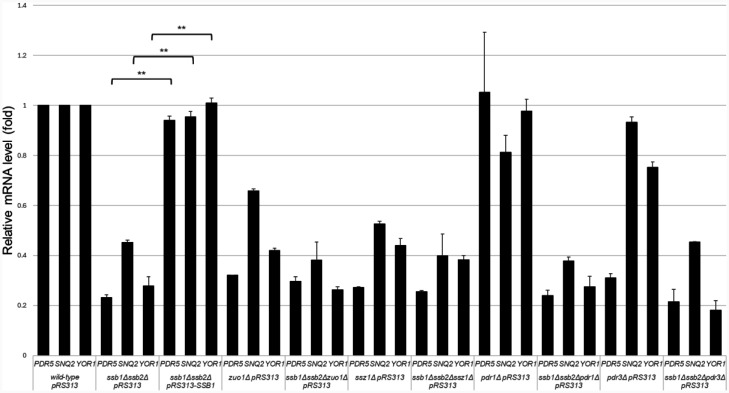
Analysis of epistatic interactions between *SSB1/2* and PDR-related genes (*ZUO1*, *SSZ1*, *PDR1*, or *PDR3*) via real-time RT-PCR. Relative mRNA levels of *PDR5, SNQ2*, and *YOR1* during the late logarithmic growth phase (OD_600_ of 1.6–1.9) were determined for each strain via real-time RT-PCR. The wild-type, *ssb1*∆*ssb2*∆, *zuo1*∆, *ssz1*∆, *pdr1*∆, *pdr3*∆, *ssb1*∆*ssb2*∆*zuo1*∆, *ssb1*∆*ssb2*∆*ssz1*∆, *ssb1*∆*ssb2*∆*pdr1*∆, and *ssb1*∆*ssb2*∆*pdr3*∆ strains transformed with an empty pRS313 plasmid are labeled wild-type pRS313, *ssb1*∆*ssb2*∆ pRS313, *zuo1*∆ pRS313, *ssz1*∆ pRS313, *pdr1*∆ pRS313, *pdr3*∆ pRS313, *ssb1*∆*ssb2*∆*zuo1*∆ pRS313, *ssb1*∆*ssb2*∆*ssz1*∆ pRS313, *ssb1*∆*ssb2*∆*pdr1*∆ pRS313, and *ssb1*∆*ssb2*∆*pdr3*∆ pRS313, respectively. The *ssb1*∆*ssb2*∆ mutant strain harboring pRS313–*SSB1* is referred to as *ssb1*∆*ssb2*∆ pRS313–*SSB1*. Two asterisks (**) indicate p values less than 0.01.

To validate whether the deletion of *SSB1/2* is responsible for the diminished mRNA levels of *PDR5, SNQ2*, and *YOR1* in the *ssb1*∆*ssb2*∆ mutant strain, we examined whether exogeneous expression of *SSB1* restores the decreased mRNA levels of *PDR5, SNQ2*, and *YOR1* in the *ssb1*∆*ssb2*∆ mutant strain during the late logarithmic growth phase (OD_600_ = 1.6–1.9). Although the mRNA levels of *PDR5, SNQ2*, and *YOR1* were significantly lower in the *ssb1*∆*ssb2*∆ pRS313 strain than in the wild-type strain (p < 0.05), the reduced mRNA levels of *PDR5, SNQ2*, and *YOR1* in the *ssb1*∆*ssb2*∆ pRS313 strain were almost completely rescued to wild-type pRS313 levels in the *ssb1*∆*ssb2*∆ pRS313–*SSB1* strain (p < 0.01) (Figure 3). The restoration of mRNA levels for *PDR5, SNQ2*, and *YOR1* suggests that the deletion of *SSB1/2* is responsible for their reduced mRNA levels in the *ssb1*∆*ssb2*∆ mutant strain.

Next, we examined whether *SSB1/2* operate via the same pathway as *ZUO1, SSZ1, PDR1*, or *PDR3*. Although the mRNA levels of *PDR5, SNQ2*, and *YOR1* were significantly lower in the *ssb1*∆*ssb2*∆ pRS313 and *zuo1*∆ pRS313 strains than in the wild-type strain (p < 0.05), *PDR5, SNQ2*, and *YOR1* mRNA levels were not additively reduced in the triple-deficient *ssb1*∆*ssb2*∆*zuo1*∆ pRS313 strain (Figure 3). In fact, ANOVA tests revealed that the mRNA levels of *PDR5, SNQ2*, and *YOR1* were not significantly different between the *ssb1*∆*ssb2*∆ pRS313, *zuo1*∆ pRS313, and *ssb1*∆*ssb2*∆*zuo1*∆ pRS313 strains (p > 0.01) (Figure 3). Thus, these data suggest that Ssb1/2 and Zuo1 function via the same pathway for transcriptional activation of *PDR5, SNQ2*, and *YOR1*. Similarly, *PDR5, SNQ2*, and *YOR1* mRNA levels were significantly lower in the *ssb1*∆*ssb2*∆ pRS313 and *ssz1*∆ pRS313 strains than in the wild-type strain (p < 0.05), whereas triple deletion of *SSB1/2* and *SSZ1* (*ssb1*∆*ssb2*∆*ssz1*∆ pRS313) did not exacerbate the reduction in *PDR5, SNQ2*, and *YOR1* mRNA levels (Figure 3). ANOVA tests also revealed that *PDR5, SNQ2*, or *YOR1* mRNA levels did not differ significantly between the *ssb1*∆*ssb2*∆ pRS313, *ssz1*∆ pRS313, and *ssb1*∆*ssb2*∆*ssz1*∆ pRS313 strains (p > 0.01). These results also suggest that Ssb1/2 and Ssz1 operate via the same pathway for transcriptional activation of ABC transporters.

However, *PDR5, SNQ2*, or *YOR1* mRNA levels did not significantly differ between the wild-type strain and the *pdr1*∆ pRS313 strain (p > 0.05) (Figure 3). These data suggest that Pdr1 is not critical for the expression of *PDR5, SNQ2*, and *YOR1*. On the other hand, the mRNA levels of *PDR5* and *YOR1* (but not those of *SNQ2*) were significantly lower in the *pdr3*∆ pRS313 strain than in the wild-type strain (p < 0.05) (Figure 3). However, the reduction levels of *YOR1* mRNA in the *pdr3*∆ pRS313 strain were small (Figure 3). These data suggest that Pdr3 is not so critical for the expression of *SNQ2* and *YOR1*. Triple deletion of *SSB1/2* and *PDR3* (*ssb1*∆*ssb2*∆*pdr3*∆ pRS313) did not exacerbate the reduction in *PDR5* mRNA levels compared with those in *ssb1*∆*ssb2*∆ pRS313 and *pdr3*∆ pRS313 (Figure 3). ANOVA tests also revealed that only the *PDR5* mRNA level was not significantly different between the *ssb1*∆*ssb2*∆ pRS313, *pdr3*∆ pRS313, and *ssb1*∆*ssb2*∆*pdr3*∆ pRS313 strains (p > 0.05) (Figure 3). These findings suggest that Ssb1/2 and Pdr3 operate via the same *PDR5* activation pathway.

### 3.4. SSB1/2 confer resistance to cycloheximide and fluconazole, possibly via the same pathway as *ZUO1*, *SSZ1*, or *PDR3*

**Figure 4.**
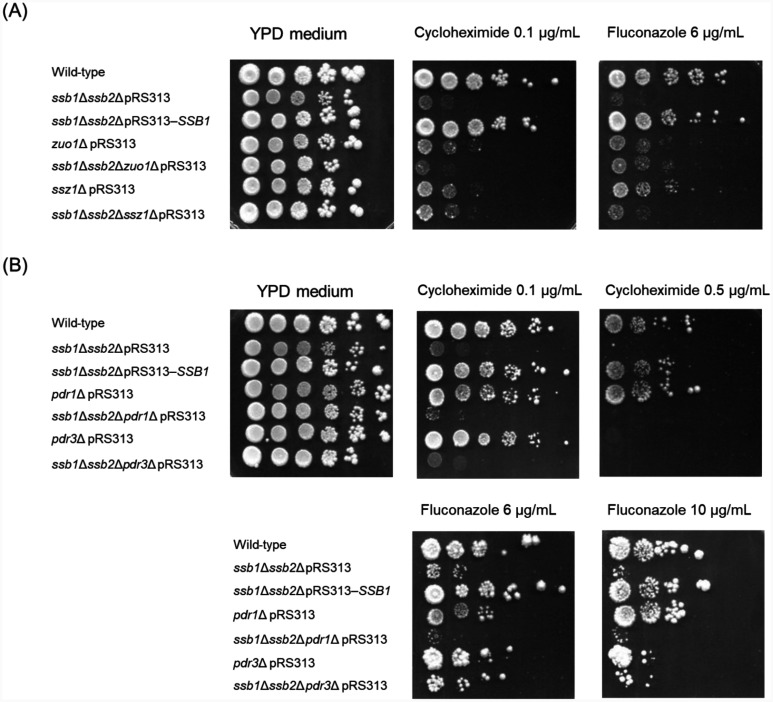
Analysis of epistatic interactions between *SSB1/2* and PDR-related genes (*ZUO1*, *SSZ1*, *PDR1*, or *PDR3*) via a spot dilution assay. (A) Resistance of the wild-type, *ssb1*∆*ssb2*∆, *zuo1*∆, *ssz1*∆, *ssb1*∆*ssb2*∆*zuo1*∆, and *ssb1*∆*ssb2*∆*ssz1*∆ strains with an empty pRS313 plasmid or pRS313–*SSB1* to fluconazole or cycloheximide was determined via a spot dilution assay. (B) Resistance of the wild-type, *ssb1*∆*ssb2*∆, *pdr1*∆, *pdr3*∆, *ssb1*∆*ssb2*∆*pdr1*∆, and *ssb1*∆*ssb2*∆*pdr3*∆ strains with an empty pRS313 plasmid or pRS313–*SSB1* to fluconazole or cycloheximide was determined via a spot dilution assay.

To determine whether *SSB1* expression from a centromere plasmid can restore resistance of the *ssb1*∆*ssb2*∆ mutant strain to fluconazole and cycloheximide, a spot dilution assay was performed. The exogenous *SSB1* expression in the *ssb1*∆*ssb2*∆ pRS313–*SSB1* strain almost completely restored resistance to cycloheximide and fluconazole in the *ssb1*∆*ssb2*∆ pRS313 strain (Figure 4A, B). The complete rescue of decreased mRNA levels of *PDR5* in the *ssb1*∆*ssb2*∆ pRS313–*SSB1* strain can explain these results, as shown in Figure 3. This rescue of susceptibility to cycloheximide and fluconazole indicates that sensitivity to cycloheximide and fluconazole in the *ssb1*∆*ssb2*∆ mutant strain results from the deletion of *SSB1/2*.

We then examined whether *SSB1/2* contribute to PDR via the same pathway as *ZUO1*, *SSZ1*, *PDR1*, or *PDR3*. As shown in Figure 4A, the *ssb1*∆*ssb2*∆ pRS313 and *zuo1*∆ pRS313 strains displayed severe susceptibility to cycloheximide and fluconazole, whereas the combined deletion in the *ssb1*∆*ssb2*∆*zuo1*∆ pRS313 strain did not have an additive effect on susceptibility to cycloheximide and fluconazole. Similarly, although the *ssb1*∆*ssb2*∆ pRS313 and *ssz1*∆ pRS313 strains exhibited severe susceptibility to cycloheximide and fluconazole, the *ssb1*∆*ssb2*∆*ssz1*∆ pRS313 strain did not have an additive susceptibility effect (Figure 4A). These data suggest that Ssb1/2, Zuo1, and Ssz1 function possibly via the same pathway for resistance to cycloheximide and fluconazole.

In contrast to the *ssb1*∆*ssb2*∆ pRS313 strain, the *pdr1*∆ pRS313 strain exhibited no susceptibility to cycloheximide or fluconazole (Figure 4B). Similar to the *ssb1*∆*ssb2*∆ pRS313 strain, the *pdr3*∆ pRS313 strain exhibited susceptibility to 0.5 µg/mL cycloheximide and 10 µg/mL fluconazole (Figure 4B). However, triple deletion of *SSB1/2* and *PDR3* did not exacerbate susceptibility to 0.1 µg/mL cycloheximide or 6 µg/mL fluconazole compared with that in the *ssb1*∆*ssb2*∆ pRS313 strain (Figure 4B). These data suggest that Ssb1/2 and Pdr3 function possibly via the same pathway for resistance to cycloheximide and fluconazole.

These differences in the susceptibility of the mutants to cycloheximide and fluconazole can also be explained by the differences in the *PDR5* mRNA levels among the strains, shown in Figure 3.

### 3.5. Addition of conditioned medium from the wild-type cells suppressed the overgrowth of ssb1∆ssb2∆ cells during diauxic shifts

The physiological roles of Ssb1/2 in the induction of ABC transporters and resistance to fluconazole and cycloheximide are currently unknown. The physiological role of the induction of *PDR5* and *SNQ2* by Zuo1 or Ssz1 involves the release of quorum-sensing molecules that cause cell growth arrest in other cells during diauxic shifts [19]. Therefore, the *zuo1*∆*ssz1*∆ and *pdr5*∆*snq2*∆ cells do not exhibit cell growth arrest during diauxic shifts and instead overgrow during diauxic shifts [19],[36]. Interestingly, the *ssb1*∆*ssb2*∆ mutant strain also exhibits overgrowth and a higher rate of budding during the diauxic shift than the wild-type strain does [8]. These results suggest that Ssb1/2 are involved in the release of quorum-sensing molecules that cause cell growth arrest via the activation of ABC transporters during the diauxic shift.

**Figure 5.**
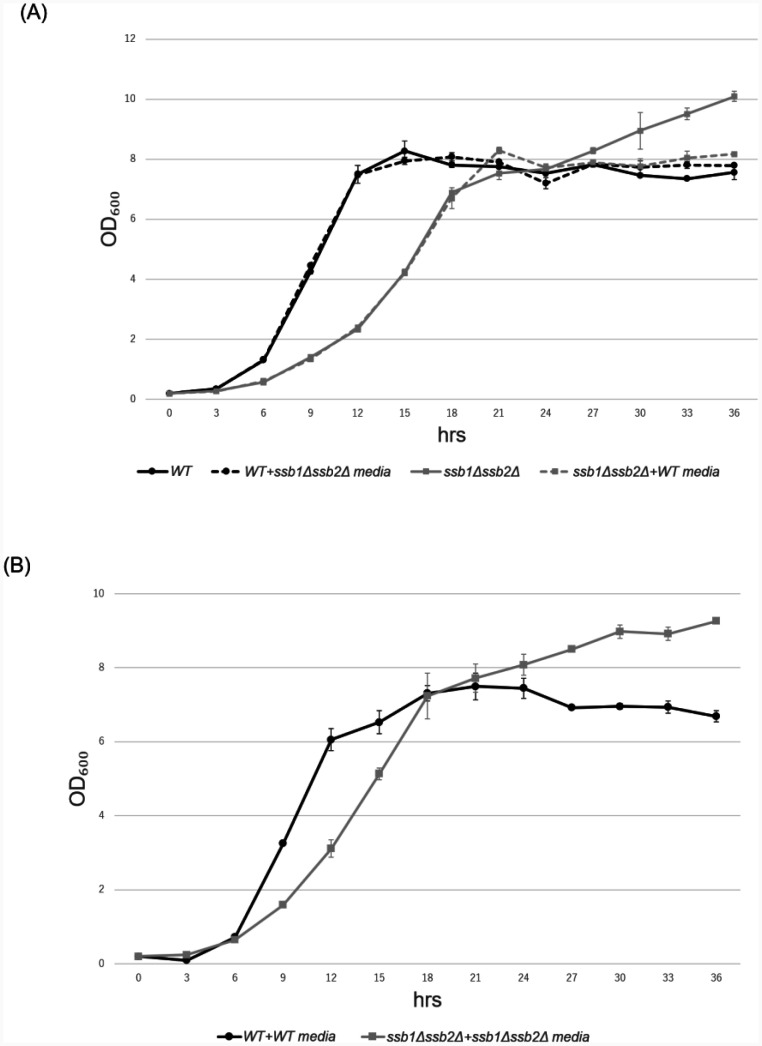
Addition of conditioned media from the wild-type strain alters the growth of *ssb1∆ssb2∆* mutant cells during the diauxic shift. (A) The black circles with solid lines represent the growth curve of the wild-type strain, and the grey squares with solid lines represent the growth curve of the *ssb1*∆*ssb2*∆ mutant strain. The black circles with dotted lines represent the growth curve of the wild-type strain, in which the culture media were replaced with conditioned media from the *ssb1*∆*ssb2*∆ mutant strain at 21 h after the start of incubation. The grey squares with dotted lines represent the growth curve of the *ssb1*∆*ssb2*∆ mutant strain, in which the culture media were replaced with conditioned media from the wild-type cells at 21 h after the start of incubation. (B) The black circles with solid lines represent the growth curve of the wild-type strain, in which the culture media were replaced with conditioned media from the wild-type strain at 21 h after the start of incubation. The grey squares with solid lines represent the growth curve of the *ssb1*∆*ssb2*∆ mutant strain, in which the culture media were replaced with conditioned media from the *ssb1*∆*ssb2*∆ mutant cells at 21 h after the start of incubation.

Thus, we examined whether conditioned medium from wild-type cells prevents overgrowth of *ssb1*∆*ssb2*∆ cells during diauxic shifts. The wild-type cells plateaued at an OD_600_ of approximately 8 after 15 h (Figure 5A). As expected, the *ssb1*∆*ssb2*∆ cells grew more slowly than the wild-type cells did during the logarithmic growth phase, but continued to grow after 27 h (Figure 5A). In addition, the overgrowth of the *ssb1*∆*ssb2*∆ cells was not suppressed when the *ssb1*∆*ssb2*∆ cells were resuspended in conditioned medium from the *ssb1*∆*ssb2*∆ cells (Figure 5B). However, the *ssb1*∆*ssb2*∆ cells plateaued at an OD_600_ of approximately 8 after 27 h, similar to the wild-type cells when resuspended in conditioned medium from the wild-type cells (Figure 5A). In contrast, when resuspended in conditioned medium from the *ssb1*∆*ssb2*∆ cells, the wild-type cells plateaued at an OD_600_ of approximately 8 after 27 h (Figure 5A).

These data suggest that Ssb1/2 are required for the release of quorum-sensing molecules that cause cell growth arrest in other cells during diauxic shifts.

## 4. Discussion

In this report, we revealed that *SSB1/2* are required for upregulating the mRNA levels of *PDR5, SNQ2*, and *YOR1* during the late logarithmic growth phase and resistance to fluconazole and cycloheximide in *S. cerevisiae*. We also showed that Ssb1/2 may act via the same pathway as Zuo1 or Ssz1 to increase *PDR5*, *SNQ2*, and *YOR1* expression and confer resistance to fluconazole and cycloheximide (Figures 3 and 4). Since Ssb1/2, Zuo1, and Ssz1 are required for the folding of nascent polypeptides, they may facilitate the proper folding of proteins that directly or indirectly regulate PDR, thereby inducing the expression of *PDR5, SNQ2*, and *YOR1*.

**Figure 6.**
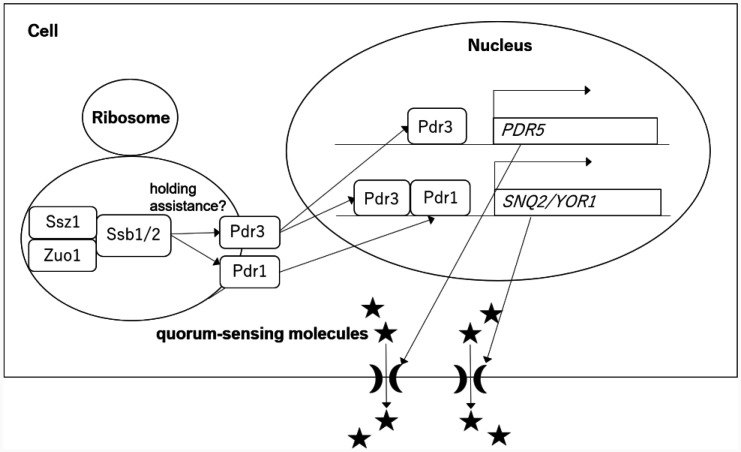
Network model mediating quorum-sensing molecule release via Ssb1/2, Zuo1, and Ssz1. In this model, Ssb1/2, Zuo1, and Ssz1 assist in the proper folding of the transcription factors Pdr1 and Pdr3, thereby enhancing the expression of the ABC transporter genes *PDR5, SNQ2*, and *Yor1*. The ABC transporters Pdr5 and Snq2 mediate the export of quorum-sensing molecules into the extracellular space.

This study also revealed that Ssb1/2 may function via the same pathway as Pdr3 (but not Pdr1) to increase *PDR5* expression and confer resistance to cycloheximide and fluconazole (Figures 3 and 4). Although *PDR5, SNQ2*, and *YOR1* are known target genes of Zuo1 and/or Ssz1, exogenous expression of the Zuo1 C-terminus or the Ssz1 N-terminus requires Pdr1 (but not Pdr3) for the activation of *PDR5* and *YOR1* expression [17]–[20]. Therefore, the loss of the molecular chaperone function in Ssb1/2, Zuo1, or Ssz1 may impair proper folding of Pdr3, leading to reduced *PDR5* expression and consequently diminished PDR (Figure 6).

Furthermore, we showed that Ssb1/2 may function via the same pathway as *ZUO1* or *SSZ1* (but not *PDR1* or *PDR3*) to increase *SNQ2* and *YOR1* expression (Figure 3). Since the transcription of *SNQ2* and *YOR1* is regulated not only by Pdr1 and Pdr3 but also by the transcription factors Yrr1 and Yrm1 [37], Ssb1/2 may assist in the folding of transcription factors other than Pdr1 and Pdr3 to increase *SNQ2* and *YOR1* expression. Alternatively, since the function of Pdr1 and Pdr3 on expression of PDR genes is redundant, Ssb1/2 may assist in the folding of both Pdr1 and Pdr3 to increase *SNQ2* and *YOR1* expression (Figure 6).

We also revealed that Ssb1/2 may be required for the release of quorum-sensing molecules that cause cell growth arrest in other cells during diauxic shifts. Ssb1/2 may increase *PDR5* and *SNQ2* transcription with Zuo1 and Ssz1 during diauxic shifts to release quorum-sensing molecules that cause cell growth arrest in other yeast cells (Figure 6). In addition, Ssb1/2 are required for the rapid inhibition of translational initiation upon glucose depletion [8]. Therefore, the dual role of the molecular chaperones Ssb1/2 in both inhibiting translational initiation and inducing cell growth arrest may link translational arrest to premature cell growth arrest during diauxic shifts.

## 5. Conclusions

Ssb1/2 increased the mRNA levels of *PDR5*, *SNQ2*, and *YOR1* during the late logarithmic growth phase and induced resistance to cycloheximide and fluconazole, possibly via the same pathway as *ZUO1* or *SSZ1*. Furthermore, Ssb1/2 induced *PDR5* during the late logarithmic growth phase and increased resistance to cycloheximide and fluconazole, possibly via the same pathway as *PDR3*. In addition, it was suggested that Ssb1/2 are required for the release of quorum-sensing molecules in culture medium, which in turn can signal cell growth arrest during diauxic shifts. This work provides useful knowledge in genetic interactions between the ribosome-associated molecular chaperone and cell growth arrest during diauxic shifts.

## Use of AI tools declaration

The authors declare they have not used Artificial Intelligence (AI) tools in the creation of this article.


